# Defining a therapeutic window for kinase inhibitors in leukemia to avoid neutropenia

**DOI:** 10.18632/oncotarget.19678

**Published:** 2017-07-28

**Authors:** Kate McArthur, Akshay A. D’Cruz, David Segal, Kurt Lackovic, Andrew F. Wilks, Joanne A. O’Donnell, Cameron J. Nowell, Motti Gerlic, David C.S. Huang, Christopher J. Burns, Ben A. Croker

**Affiliations:** ^1^ Walter and Eliza Hall Institute of Medical Research, Melbourne, VIC, Australia; ^2^ Department of Medical Biology, University of Melbourne, Melbourne, VIC, Australia; ^3^ Division of Hematology/Oncology, Boston Children's Hospital, Boston, MA, USA; ^4^ Department of Pediatrics, Harvard Medical School, Boston, MA, USA; ^5^ Department of Molecular, Cell and Cancer Biology, University of Massachusetts Medical School, Worcester, MA, USA; ^6^ Monash Institute of Pharmaceutical Sciences, Melbourne, VIC, Australia; ^7^ Department of Clinical Microbiology and Immunology, Tel Aviv University, Tel Aviv, Israel; ^8^ School of Chemistry, Bio21, The University of Melbourne, Melbourne, VIC, Australia

**Keywords:** apoptosis, neutropenia, kinase inhibitors, hematopoietic progenitor cells, leukemia

## Abstract

Neutropenia represents one of the major dose-limiting toxicities of many current cancer therapies. To circumvent the off-target effects of cytotoxic chemotherapeutics, kinase inhibitors are increasingly being used as an adjunct therapy to target leukemia. In this study, we conducted a screen of leukemic cell lines in parallel with primary neutrophils to identify kinase inhibitors with the capacity to induce apoptosis of myeloid and lymphoid cell lines whilst sparing primary mouse and human neutrophils. We have utilized a high-throughput live cell imaging platform to demonstrate that cytotoxic drugs have limited effects on neutrophil viability but are toxic to hematopoietic progenitor cells, with the exception of the topoisomerase I inhibitor SN-38. The parallel screening of kinase inhibitors revealed that mouse and human neutrophil viability is dependent on cyclin-dependent kinase (CDK) activity but surprisingly only partially dependent on PI3 kinase and JAK/STAT signaling, revealing dominant pathways contributing to neutrophil viability. Mcl-1 haploinsufficiency sensitized neutrophils to CDK inhibition, demonstrating that Mcl-1 is a direct target for CDK inhibitors. This study reveals a therapeutic window for the kinase inhibitors BEZ235, BMS-3, AZD7762, and (R)-BI-2536 to induce apoptosis of leukemia cell lines whilst maintaining immunocompetence and hemostasis.

## INTRODUCTION

Acute Myeloid Leukaemia (AML) is a heterogeneous disease characterised by an abundance of mutations in genes encoding signalling proteins, transcription factors, tumor suppressors and chromatin modifying proteins [1]. Cytogenetic profiling and molecular genetic analysis is used to stratify patients into low, intermediate and high-risk groups, and whilst low-risk groups respond well to conventional cytotoxic chemotherapy, the majority of patients do not [2]. Indeed, less than 40% of adults with AML are cured, with the situation being particularly poor for the elderly who often have co-morbid conditions, where median survival is approximately 10 months [3]. Against this backdrop there has been a concerted effort over the last decade to develop targeted therapies for AML, with the aim to target specific genetic lesions driving oncogenesis [4]. In particular, there has been a considerable focus on the development of selective kinase inhibitors for the treatment of AML, such as FLT3 inhibitors for the ~30% of patients who possess activating FLT3 mutations [5, 6] .

Targeted therapies have also been proposed [7] for the treatment of T cell acute lymphoblastic leukemias (T-ALLs). T-ALL displays a variety of genetic complexities arising from mutations in signalling proteins, transcription factors and tumor suppressors. BCR-ABL inhibitors such as imatinib, nilotinib and dasatinib may be of use to ALL patients with ABL fusion proteins [8].

Of the hematological toxicities associated with chemotherapy regimes, neutropenia is the most common dose-limiting side-effect [9–12]. Neutropenia renders the immune system incapable of effective responses to many types of pathogens, and the duration and severity is strongly linked to morbidity and mortality [9], with average mortality being shorter for patients with hematological malignancies compared to those with solid tumors [13]. In addition neutropenia is associated with a significant economic burden due to treatment and hospitalization [13].

To better understand the clinical potential of targeted therapies in leukemia and particularly AML, we have screened a library of 95 known and well-studied kinase inhibitors and examined the apoptosis-inducing effect of these compounds against a panel of leukemia lines. In conjunction, we have used a live-cell imaging platform previously developed by us to determine if these compounds also induce apoptosis of neutrophils and hematopoietic progenitor cells, as a method of assessing their potential to cause neutropenia. With this combined experimental design we have identified individual kinase inhibitors that provide a therapeutic window for the effective treatment of leukemias with minimal effects on neutrophil viability. In addition, the data obtained in our neutrophil viability screen has identified kinase targets whose inhibition significantly impacts neutrophil survival via effects on Mcl-1 and may contribute directly to neutropenia *in vivo*.

## RESULTS

### Effect of compounds on leukemia cell proliferation

A library of 95 discrete kinase inhibitors was established with activity across all kinase families in the human kinome (Table 1). All compounds in the collection have been studied extensively with broad kinome selectivity data for most compounds available in the literature [18–20]. Many of the inhibitors are used therapeutically or are in advanced clinical development. In addition, the BH3 mimetic ABT-737 [21], a compound with potent activity against leukemias [22], was included as a control compound.

**Table 1:** Compound and reported target

Compound	Reported Targets	Compound	Reported Targets	Compound	Reported Targets
**ABT-737**	Bcl-2 proteins	**CYC-116**	Aurora	**PI103**	PI3K p110α
**ABT-869**	RTK	**CYT387**	JAK1, JAK2, TYK2	**PIK-294**	PI3K p110α
**AC220**	FLT3, FMS, cKIT, PDGFR	**Danusertib**	AUR, BCR-ABL, FGFR	**PIK-75**	PI3K p110α, CDK7, CDK9
**AG13958**	VEGF	**Dasatinib**	AUR, BCR-ABL, FGFR	**PIK-90**	PI3K p110α
**Akt-I-1**	Akt1	**E7080**	VEGFR, FGFR, SCFR	**PLX4720**	B-RafV600E
**Akt-I-1,2**	Akt1/2	**Erlotinib**	EGFR	**Purvalanol-B**	CDK
**AMG-47a**	Lck	**Flavopiridol**	CDKs	**R1487**	p38 MAPK
**AMG-Tie2-1**	TIE-2	**GDC-0941**	PI3K	**RHO-15**	ROCK1
**AS-252424**	PI3K p110γ	**Gefitinib**	EGFR	**RWJ-67657**	p38MAPK
**AT-7519**	CDKs	**GSK690693**	Akt1, Akt2 and Akt3	**SB202190**	p38 MAPK
**AT9283**	Aurora	**GW441756**	TrkA	**SB203580**	p38 MAPK
**AV-412**	EGFR	**IC87114**	PI3K p110δ	**SB216763**	GSK3
**AV951**	VEGFR	**Imatinib**	BCR-ABL	**SB242235**	p38 MAP
**Axitinib**	VEGF, PDGF, c-Kit	**JNJ38877605**	c-MET	**SB590885**	B-Raf
**AZ-960**	JAK1, JAK2, JAK3	**JNJ7706621**	CDK	**SD-06**	p38MAPK
**AZD1480**	JAK2	**Ki20227**	cFMS	**SD169**	p38α MAP kinase
**AZD6244**	MEK	**KU0063794**	mTOR	**SNS-032**	CDK2, CDK7, CDK9
**AZD7762**	CHK	**KU55933**	ATM	**SNS-314**	Aurora
**BEZ-235**	PI3K, mTOR	**KU57788**	DNA-PK	**Sorafenib**	Raf & VEGFR
**(R)-BI-2536**	Plk1	**Lapatinib**	EGFR, ErbB-2	**SR3677**	ROCK-II
**BI-D1870**	RSK	**Masatinib**	c-Kit	**SU-5402**	FGFR, c-Kit
**Bisindoylmaleimide X**	PKC	**Merck 5**	JAK1, JAK2, JAK3, TYK2	**SU-6668**	VEGF, PDGFR and FGFR
**BMS-3**	LIMK	**Motesanib**	c-Kit, PDGFR, VEGFR	**Sunitinib**	cFMS, Flt3,Kit, PDGFR, RET & VEGFR
**BMS-5**	LIMK	**MP-470**	Flt3,Met, Kit,c-Ret, PDGFR	**TAK-715**	p38 MAPK
**Bosutinib**	Bcr-Abl, Src	**Nilotinib**	BCR-ABL, cKit, PDGFR	**Tandutinib**	FLT3, PDGF, c-Kit
**BX795**	PDK1, TBK1	**Pazopanib**	VEGF	**TGX221**	PI3-K p110β
**BX912**	PDK1	**PD0325901**	MEK	**TOVOK**	EGFR, HER2
**CC-401**	JNK	**PF-562271**	FAK	**Vandetanib**	VEGFR, EGFR
**CI-1033**	EGFR	**PF04217903**	c-Met	**Vargatef**	VEGF, PDGFR, FGFR
**CI-1040**	MEK1, MKK5	**PF431396**	PYK2	**VX-680**	Aurora
**CP-724714**	ErbB2	**PHA690509**	CDK-A	**VX702**	p38 MAPK
**CP690550**	JAK1, JAK2, JAK3	**PI-93**	PI3K p110α	**YM201636**	PIK5

**Figure 1:**
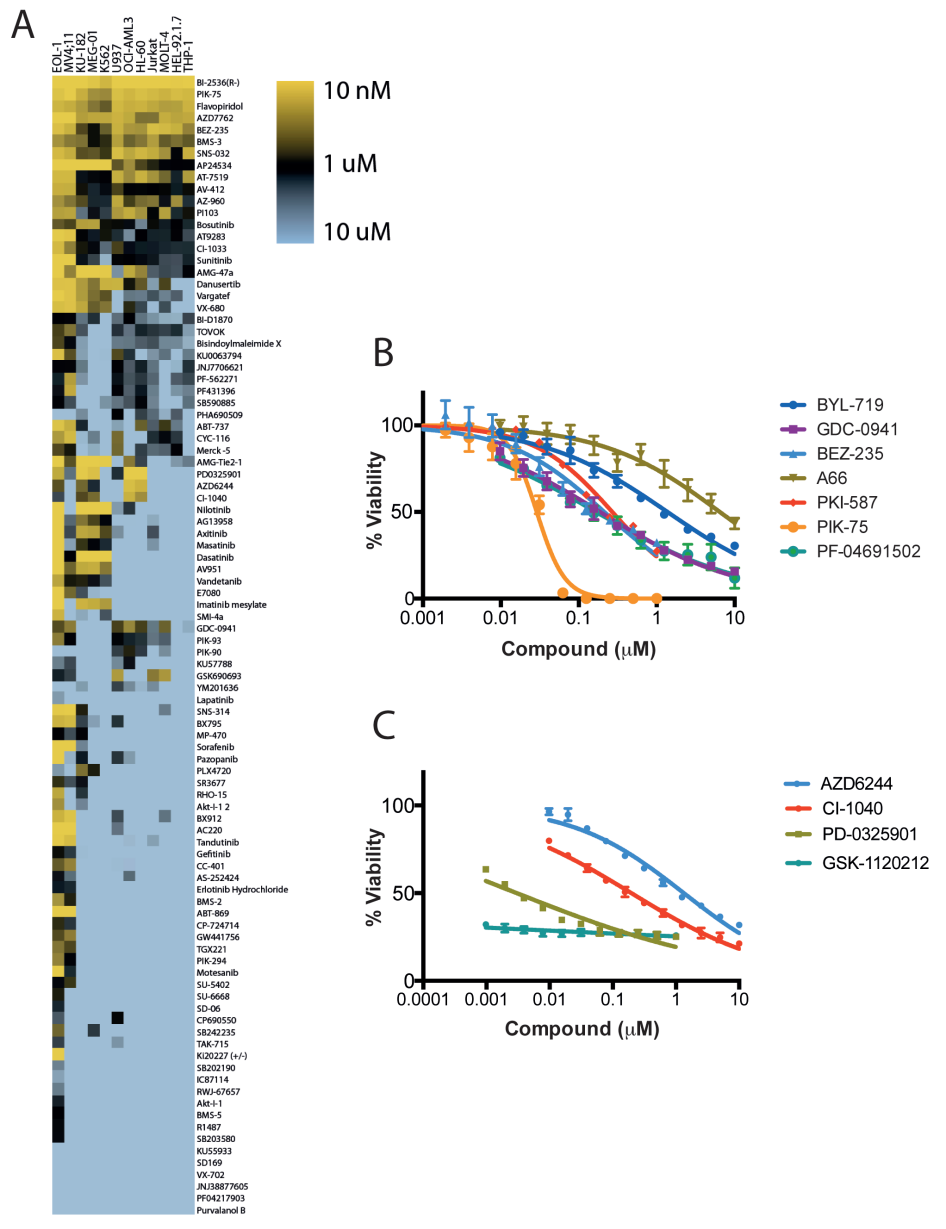
Effects of kinase inhibition on the proliferation of leukemia cells **A**. Human leukemia cell lines were treated with duplicate serial dilutions of kinase inhibitors and the effect on cell viability was determined by Cell Titer Glo assay after 48 hours. Data shown represents the mean IC50 of each condition from 2 independent experiments. HL-60 cells were treated with serial dilutions of mTor/PI3K **B**. and MEK **C**. inhibitors and the effect on cell viability was determined after 48 hours. Data shown is the mean ± SEM of 3 independent experiments. Note, in some instances error bars are smaller than symbols displayed.

The activity of the kinase inhibitor collection against a panel of human leukemia cell lines was determined in cell viability assays run over 48 h using the Cell Titer Glo reagent. The cell lines examined consisted of five AML lines (MV4;11, HL-60, OCI-AML3, THP-1, U937), two chronic myeloid leukemia (CML) lines (K562 and KU-812), two T-cell leukemia lines (MOLT4, JURKAT), and three additional leukemia lines (EOL-1, HEL.92.1.7, MEG-01), representing eosinophilic leukemia, erythroleukemia and megakaryoblastic leukemia, respectively. Compounds in this collection displayed a wide diversity of responses across the panel of cell lines (Figure 1A, Table 2). A substantial number of compounds showed potent activity against both MV4;11 and EOL-1 cell lines, whereas other cell lines (HEL 92.1.7, THP-1 and MOLT-4) appeared largely resistant to effects on cellular viability over the timeframe of the experiment. A small number of compounds showed potent activity (IC_50_ ≤ 10 nM) across most of the cell lines examined. These pan-active compounds consist of the first-generation PI3K p110α (PI3Kα) inhibitor PIK-75 [23], the PLK1 inhibitor BI-2536 [24], the PI3K/mTOR inhibitor BEZ-235 [25], and the Chk1/2 inhibitor AZD-7762 [26]. Further study of a panel of reported PI3K/mTOR inhibitors in HL60 cells confirmed the results from the preliminary screen, with all compounds, including the clinical agent PKI-587, PF-04691502 and GDC-0941 possessing significant anti-proliferative activity (Figure 1B). Notably, compounds with selectivity for members of the PI3K family were significantly weaker in this assay (Figure 1B). Compounds possessing MEK inhibitory activity (PD-0325901, CI-1040) possessed potent activity selectively against the HL-60 cell line, further confirmed on repeat dose-response studies with the inclusion of the highly potent MEK inhibitors GSK-1120212 and PD-0325901 (Figure 1C).

**Table 2:** IC50 of inhibitors on cell lines

Drug Name	m	SD	m	SD	m	SD	m	SD	m	SD	m	SD	m	SD	m	SD	m	SD	m	SD	m	SD	m	SD
ABT-737	0.05	0.03	3.52	0.04	0.53	0.23	2.91	0.2	10	0	1.88	0.03	6.12	5.08	0.71	0.32	0.14	0.03	6.2	1.31	10	0	4.41	1.45
ABT-869	0.01	0	10	0	10	0	5.22	6.76	9.49	0.45	10	0	10	0	10	0	0.01	0	10	0	10	0	10	0
AC220	0	0	9.13	1.23	10	0	6.12	5.49	10	0	9.86	0.2	6.33	5.19	9.88	0.18	0.01	0.01	10	0	10	0	3.36	2.6
AG13958	0	0	6.26	0.22	6.2	1.48	4.55	0.29	1.37	0.09	0.43	0.11	0.66	0.14	6.75	0.71	3.13	0.68	4.98	0.63	9.71	0.41	5.55	0.15
Akt-I-1	1.82	1.09	10	0	10	0	10	0	10	0	10	0	10	0	10	0	8.48	2.5	10	0	10	0	10	0
Akt-I-1,2	0.3	0.11	10	0	9.88	0.17	8.45	0.46	9.62	0.66	3.81	0.3	6.33	0.22	8.38	2.3	6.8	3.69	9.59	0.58	10	0	9.64	0.51
AMG-47a	0	0	2.7	0.32	0.34	0.08	2.93	1.26	0.02	0.01	0.01	0.01	0.01	0	2.5	1.41	0.36	0.07	0.17	0.07	1.3	0.04	4.03	0.3
AMG-Tie2-1	0	0.01	4.18	1.3	2.41	0.42	9.65	0.5	0.02	0.01	0.01	0	0.01	0	9.49	0.72	0.6	0.18	0.41	0.13	3.56	0.56	5.12	0.35
AP24534	0	0	1.18	0.03	0.65	0.11	0.82	0.15	0	0	0	0	0.01	0.01	1.38	1.12	0.01	0.01	0.3	0.02	1.21	0.62	0.58	0.11
AS-252424	1.53	0.49	10	0	10	0	10	0	9.58	0.72	6.02	0.4	10	0	10	0	3.26	1.74	3.18	0.56	10	0	8.01	0.08
AT-7519	0.1	0.01	1.79	0	0.14	0.02	0.27	0.01	1.18	0.23	1.56	0.36	1.41	0.44	0.49	0.1	0.1	0.02	0.32	0.05	0.24	0.07	0.15	0.03
AT9283	0.02	0.02	0.98	0.23	1.03	0.06	1.81	0.44	0.87	0.16	0.95	0.13	1.72	0.3	1.83	0.38	0.03	0.01	3.95	0.16	2.56	0.59	1.98	0.02
AV-412	0.14	0.1	1.69	0.1	0.95	0.37	1	0.35	1.48	0.4	0.87	0.01	1.88	1.39	0.93	0.16	0.15	0	1.21	0.17	0.91	0.03	0.66	0.07
AV951	0	0	10	0	7.66	0.72	10	0	0.41	0.13	0.17	0.02	0.15	0.07	9.1	1.28	0.52	0.07	10	0	10	0	9.02	1.39
Axitinib	0	0	7.87	3.02	6.09	5.54	2.62	1.56	0.64	0.16	0.19	0.01	0.21	0.02	7.43	3.63	2.5	0.98	8.89	1.57	10	0	3.97	3.56
AZ-960	0.29	0.23	0.29	0.01	0.48	0.1	0.43	0.05	1.92	0.67	0.62	0.05	1.86	1.49	1.51	0.31	0.43	0	0.69	0.04	2.03	1.97	0.17	0.03
AZD6244	0.6	0.53	10	0	0.12	0.02	10	0	7.91	2.85	0.5	0.03	1.96	0.82	10	0	5.24	1.62	0.07	0.02	7.01	0.46	9.18	1.16
AZD7762	0	0	0.2	0.05	0.45	0.05	0.44	0.08	0.18	0.09	0.23	0	0.18	0.1	0.15	0.12	0.01	0.01	0.17	0.02	0.17	0.02	0.12	0.01
BEZ-235	0.06	0.05	0.14	0.08	0.27	0.13	0.07	0.03	0.54	0.28	0.65	0.07	0.89	0.57	0.09	0.03	0.14	0.06	0.26	0.04	0.35	0.34	0.13	0.04
(R)-BI-2536	0.01	0	0.02	0.01	0.01	0	0.01	0	0.1	0.13	0.01	0	0.04	0.05	0.01	0	0.01	0	0.01	0	0.01	0	0.01	0.01
BI-D1870	0.99	0.71	4.7	1.74	3.36	1.36	4.07	1.72	8.13	1.73	3.37	0.17	2.03	1.28	4.61	0.37	1.25	0.2	1.02	0.26	2.4	0.17	3.29	0.45
Bisindoylmaleimide X	0.65	0.36	3.83	0.56	2.91	1.17	2.64	0.28	6.04	0.83	4.34	0.14	5.63	4.25	3.82	1.42	1.45	0.72	3.44	0.02	4.67	1.26	3.53	0.11
BMS-2	0.29	0.07	10	0	9.43	0.81	10	0	7.93	2	7.63	2.7	5.87	4.57	10	0	0.82	0.25	8.88	1.58	9.74	0.37	10	0
BMS-3	0.28	0.22	0.46	0.12	0.42	0.07	0.23	0.05	0.78	0.28	0.45	0.03	0.9	0.91	0.55	0.02	0.4	0.02	0.52	0.12	0.56	0.12	0.22	0.02
BMS-5	1.07	1.15	10	0	10	0	10	0	10	0	10	0	10	0	10	0	10	0	10	0	10	0	10	0
Bosutinib	0.56	0.05	1.01	0.26	3.73	3.69	0.91	0.14	0.88	0.17	0.23	0.02	0.21	0.07	1.7	1.79	0.66	0.07	1.48	0.08	1.89	0.1	0.91	0.52
BX795	0.12	0.08	5.43	1.66	10	0	9.08	1.3	9.27	0.64	2.38	0.1	4.41	1.83	10	0	0.1	0.05	10	0	10	0	2.07	0.43
BX912	0.12	0.1	10	0	8.47	2.16	7.57	0.15	7.13	3.5	9.63	0.53	7.25	3.89	3.37	2.25	0.05	0.01	10	0	10	0	2.57	0.14
CC-401	0.55	0.34	10	0	9.24	1.08	10	0	10	0	8.34	0.61	10	0	10	0	0.35	0.06	4.93	1.47	10	0	4.93	2.58
CI-1033	0.09	0.01	1.97	0.14	1.69	0.9	1.73	0.41	3.22	0.8	0.86	0.12	1.43	0.1	1.87	0.41	0.37	0.03	1.51	0.13	1.93	0.19	0.67	0.04
CI-1040	2.52	1.99	10	0	0.3	0.08	10	0	9.13	0.76	0.64	0.5	0.49	0.41	9.86	0.19	6.33	4.25	0.13	0.06	10	0	7.3	3.23
CP-724714	0.78	0.63	10	0	10	0	10	0	10	0	6.94	4.32	10	0	10	0	2.14	0.32	10	0	10	0	10	0
CP690550	2.81	2.52	7.83	3.06	10	0	10	0	10	0	10	0	5	7.07	10	0	9.34	1.32	10	0	10	0	0.94	0.4
CYC-116	0.26	0.22	2.18	0.67	6.25	4.37	1.97	1.97	8.46	0.7	4.91	0.99	3.61	2.09	1.44	0.04	0.09	0.04	7.67	2.53	8.96	1.47	0.48	0.04
Danusertib	0.05	0.01	5.75	0.97	0.56	0.06	4.32	0.25	0.14	0.06	0.24	0.04	0.43	0.16	0.66	0.38	0.04	0.01	0.87	0.38	10	0	0.13	0.17
Dasatinib	0.01	0	9.86	0.2	10	0	7.55	2.54	0	0	0	0	0.01	0.01	6.75	4.59	1.08	0.92	8.26	0.21	10	0	9.33	0.95
E7080	0	0.01	7.44	3.62	7.5	3.54	10	0	5.79	1.38	2.66	0.89	4.82	1.63	10	0	0.5	0.18	10	0	10	0	10	0
Erlotinib	1.68	1.45	8.52	0.26	9.26	1.04	6.73	0.12	10	0	5.48	0.02	5.68	0.11	10	0	1.9	0.21	10	0	10	0	10	0
Flavopiridol	0.11	0.03	0.22	0.01	0.12	0	0.18	0.01	0.55	0.13	0.25	0.01	0.42	0.16	0.23	0.03	0.15	0.03	0.14	0.02	0.13	0.02	0.1	0.01
GDC-0941	0.62	0.42	10	0	0.68	0.64	3.46	0.22	8.39	2.8	10	0	9.1	1.28	0.52	0.33	0.39	0.11	0.34	0.02	4.64	4.6	0.67	0.26
Gefitinib	0.9	0.61	9.42	0.82	10	0	5.59	2.16	10	0	4.96	0.72	9.1	1.27	8.34	2.34	2.22	0.71	7.32	1.61	10	0	5.53	2
GSK690693	1.75	1.18	10	0	6.3	0.21	0.41	0.16	10	0	10	0	10	0	0.26	0.17	2.35	0.68	10	0	10	0	0.25	0.01
GW441756	0.62	0.7	10	0	10	0	10	0	7.41	4.49	10	0	10	0	10	0	0.38	0.16	10	0	10	0	10	0
IC87114	4.43	3.95	10	0	10	0	10	0	10	0	10	0	10	0	10	0	5.77	1.75	10	0	10	0	5.09	6.94
Imatinib	0	0	10	0	10	0	9.55	0.64	0.25	0.12	0.12	0	0.16	0.08	10	0	8.82	1.71	10	0	10	0	10	0
JNJ38877605	7.43	4.8	10	0	10	0	10	0	10	0	10	0	10	0	10	0	10	0	10	0	10	0	10	0
JNJ7706621	1.32	0.23	4.8	0.34	1.76	0.58	3.39	0.17	5.23	1.01	3.48	0.27	5.91	0.92	7.81	1.79	1.28	0.22	2.86	0.44	2.05	0	1.09	0.4
Ki20227	0	0	10	0	10	0	10	0	10	0	9.44	0.79	10	0	10	0	6.62	1.51	10	0	10	0	5.25	0.76
KU0063794	0.06	0.04	5	2.08	4.97	0.12	4.12	2.01	4.94	1.73	4.39	0.55	5.12	3.07	3.32	0.01	0.61	0.1	2.04	0.62	3.54	2.97	0.63	0.18
KU55933	6.97	3.84	10	0	8.13	2.64	10	0	10	0	10	0	10	0	10	0	7.31	3.11	10	0	10	0	7.79	2.49
KU57788	3.6	1.94	8.94	0.38	6.2	4.55	9.14	1.21	10	0	10	0	7.44	1.58	9.93	0.1	2.52	2	1.51	0.35	6.59	4.83	3.87	1.84
Lapatinib	4.25	4.23	9.37	0.89	6.91	1.03	6.86	0.35	9.54	0.5	5.01	2.74	10	0	9.08	1.29	9.92	0.16	6.41	2.57	9.53	0.4	6.13	2.17
Masatinib	0	0	10	0	7.64	3.34	4.11	1.23	1.95	0.46	0.74	0.08	1.38	0.28	8.41	2.24	4.43	0.74	5.7	0.28	9.64	0.43	7.99	1.98
Merck -5	0.64	0.24	0.98	0.23	2.3	1.04	3.17	1.17	9.61	0.67	10	0	4.61	0.59	3	1.06	0.85	0.22	9.49	0.73	7.26	3.87	0.5	0.12
Motesanib	0.01	0	10	0	10	0	10	0	10	0	10	0	10	0	10	0	1.89	0.54	10	0	10	0	10	0
MP-470	1.11	0.61	5.12	6.91	10	0	10	0	9.09	1.58	1.2	0.92	5.29	1.27	10	0	2.6	1.29	6.2	4.43	10	0	6.75	4.6
Nilotinib	0	0	7.71	0.72	5.39	3.78	5.76	0.21	0.01	0	0.01	0	0.01	0	10	0	6.83	3.75	2.13	0.14	7.55	3.47	4.25	0.16
Pazopanib	0.02	0	10	0	9.48	0.74	10	0	9.11	1.54	1.68	0.09	5.55	1.85	10	0	4.73	3.62	4.24	0.43	10	0	2.11	0.04
PD0325901	0.17	0.05	10	0	0	0	10	0	10	0	0.02	0	0.06	0.03	10	0	3.34	4.63	0.01	0	5.82	5.92	2	1.4
PF-562271	1.79	0.41	2.88	0.87	2.19	0.11	3.1	0.21	7.64	0.45	4.12	0.68	6.62	0.71	6	0.59	0.22	0.03	2.72	0.36	2.47	0.55	1.57	0.35
PF04217903	8.02	2.31	10	0	10	0	10	0	10	0	10	0	10	0	10	0	10	0	10	0	10	0	10	0
PF431396	1.52	0.36	4.07	0.74	1.64	0.05	3.61	0.18	7.1	2.25	5.24	0.28	6.32	0.36	6.42	1.28	0.16	0.03	2.96	0.08	3.66	1.69	1.23	0.14
PHA690509	5.31	5.04	2.88	0.75	2.35	0.05	3.21	0.73	5.98	0.8	3.93	0.3	5.39	1.07	4.68	1.69	5.89	4.75	6.23	0.42	5.98	0.88	1.05	0.13
PI103	0.22	0.05	1.89	1.2	0.29	0.07	0.96	0.12	2.29	0.08	3.35	3.04	1.52	0.24	0.21	0.01	0.2	0.09	0.16	0	0.88	0.53	0.26	0.04
PIK-294	0.36	0.15	10	0	10	0	10	0	10	0	10	0	10	0	10	0	1.3	0.72	9.4	0.86	10	0	10	0
PIK-75	0.04	0.02	0.08	0	0.06	0.01	0.09	0	0.33	0.11	0.14	0.02	0.29	0.07	0.13	0.01	0.01	0	0.1	0.01	0.1	0.04	0.06	0.01
PIK-90	5.09	3.94	10	0	2.24	1.3	9.47	0.75	10	0	10	0	10	0	3.92	0.95	6.41	3.61	0.82	0.47	5.41	4.63	2.32	1.95
PIK-93	0.39	0.39	8.14	0.67	2.07	1.32	3.4	0.54	9.98	0.03	10	0	10	0	2.95	1.27	1.23	0.32	1.8	0.14	7.05	4.06	1.29	0.69
PLX4720	4.2	0.49	10	0	10	0	9.68	0.46	10	0	0.45	0.06	0.84	0.48	10	0	5.6	5.09	10	0	10	0	8.5	0.77
Purvalanol B	10	0	10	0	10	0	10	0	10	0	10	0	10	0	10	0	9.13	1.74	10	0	10	0	10	0
R1487	1.41	1.13	10	0	10	0	10	0	10	0	10	0	10	0	10	0	10	0	10	0	10	0	10	0
RHO-15	0.19	0.13	10	0	10	0	10	0	7.75	3.9	2.11	0.47	5.14	3.3	10	0	7.22	3.23	10	0	10	0	9.64	0.51
RWJ-67657	3.59	4.34	10	0	10	0	10	0	10	0	5.77	0.54	10	0	10	0	8.28	2.02	10	0	10	0	8.05	2.76
SB202190	3.68	2.38	10	0	10	0	10	0	10	0	9.36	0.91	6.83	4.48	10	0	7	3.12	10	0	10	0	6.6	4.81
SB203580	1.45	1.92	10	0	10	0	10	0	10	0	10	0	10	0	10	0	10	0	10	0	10	0	10	0
SB242235	0.51	0.21	10	0	10	0	10	0	10	0	10	0	2.18	0.64	10	0	6.16	2.66	10	0	10	0	10	0
SB590885	1.3	0.2	5.26	0.61	1.41	0.01	3.76	1.06	4.06	0.9	5.01	0.16	5.04	0.39	5.93	0.52	2.59	0.06	2.71	0.07	3.31	0.12	3.42	0.03
SD-06	1.87	2.26	10	0	10	0	10	0	10	0	10	0	10	0	5.03	7.03	5.67	5	10	0	10	0	10	0
SD169	5.12	5.64	10	0	10	0	10	0	10	0	10	0	10	0	10	0	10	0	10	0	10	0	10	0
SMI-4a	0.13	0.07	10	0	10	0	10	0	2.74	1.72	4.54	0.53	5.78	0.69	10	0	9.71	0.58	10	0	10	0	10	0
SNS-032	0.09	0.03	1.33	0.48	0.1	0.04	0.16	0.05	0.72	0.24	0.61	0.3	0.58	0.28	0.29	0.09	0.15	0.04	0.19	0.01	0.14	0.03	0.07	0.01
SNS-314	0.02	0	10	0	6.62	4.39	9.01	1.4	9.11	1.54	0.83	0.25	8.15	2.62	3.58	5.06	0.01	0	10	0	7.37	3.73	5.04	1.83
Sorafenib	0	0	10	0	5.73	0.91	10	0	9.94	0.1	3.79	2.84	6.28	5.26	10	0	0.01	0	5.46	1.56	10	0	5.33	2.86
SR3677	0.75	0.1	10	0	9.1	1.27	10	0	10	0	1.53	0.56	8.05	2.76	10	0	2.64	1.76	10	0	10	0	5.96	0.21
SU-5402	1.43	0.45	10	0	10	0	10	0	10	0	9.28	1.02	10	0	10	0	0.73	0.26	10	0	10	0	10	0
SU-6668	0.84	0.43	10	0	10	0	10	0	8.11	3.28	10	0	10	0	10	0	5.11	2.22	10	0	10	0	10	0
Sunitinib	0.01	0	2.61	0.01	1.46	0.14	1.62	0.33	4.75	0.62	1.56	0.68	2.39	0.43	2.54	0.33	0.01	0	1.02	0.24	1.8	0.93	1.16	0.81
TAK-715	2.33	0.65	10	0	10	0	9.58	0.54	7.41	2.38	6.14	1.4	7.37	3.38	10	0	7.8	2.57	10	0	10	0	4.11	1.34
Tandutinib	0.02	0	10	0	7.58	2.75	4.38	0.24	8.85	1.13	6.6	0.46	7.91	2.95	9.77	0.33	0.08	0.02	4.87	0.73	7.42	0.15	7.44	0.12
TGX221	0.43	0.31	10	0	10	0	9.84	0.22	9.16	0.82	10	0	10	0	10	0	0.65	0.37	9.72	0.39	10	0	9.85	0.21
TOVOK	0.68	0.23	1.94	0.23	1.8	0.34	2.11	0.07	5.72	0.57	2.71	0.28	5.02	4.75	2.37	0.61	0.44	0.08	3.36	1.17	4.31	1.68	2.06	0.86
Vandetanib	0.19	0.07	10	0	7.28	1.77	6.28	0.44	2.73	0.73	0.82	0.05	1.51	0.25	6.79	1.8	0.86	0.16	10	0	10	0	9.46	0.76
Vargatef	0	0.01	2.97	0.05	3.55	1.13	2.7	0.11	0.59	0.36	0.24	0.02	0.46	0.03	3.69	1.49	0.04	0	3.44	1.29	5.85	0.32	6.31	1.13
VX-680	0.07	0.02	7.99	0.45	2.45	1.52	4.96	0.49	0.43	0.08	0.18	0.02	0.53	0.11	2.59	0.88	0.05	0	0.8	0.03	5.75	6.01	5.46	5.21
VX702	5.5	2.69	10	0	10	0	10	0	10	0	10	0	10	0	10	0	10	0	10	0	10	0	10	0
YM201636	5.88	4.76	7.14	1.61	4.82	1.92	3.94	0.59	9.65	0.31	4.28	0.14	9.12	1.24	6.99	0.19	7.33	3.11	3.88	0.02	8.78	1.72	2.39	0.41

Values are shown in uM. For values less than 10 nM, they are represented as 0 uM. m:mean; SD:standard deviation.

### Effect of compounds on neutrophil survival

**Figure 2:**
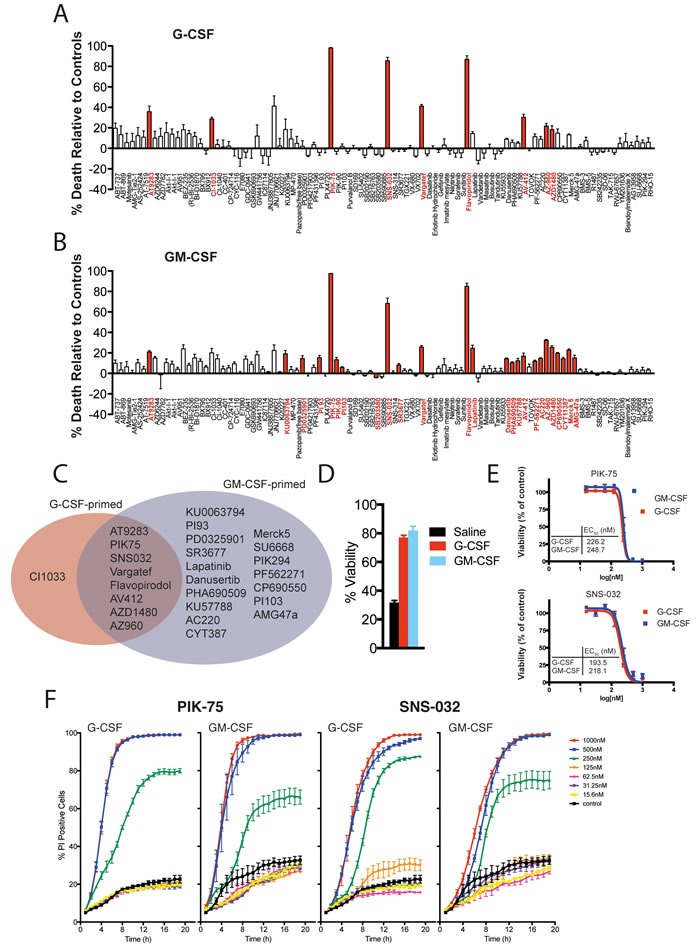
Viability of neutrophils in response to kinase inhibitors **A**. & **B**. Viability analysis from live cell imaging of primary murine bone-marrow neutrophils primed with either G-CSF (A) or GM-CSF (B) in response to a panel of kinase inhibitors [μM] over 24 hours. Bar graphs display percentage viability relative to G-CSF- or GM-CSF-only primed controls at 12 hours. Red bars denote significance, *p* < 0.05 (corrected by Hommel's modification of a Bonferroni t test). **C**. Summary of data in A) & B) highlighting the kinase inhibitors that significantly altered neutrophil viability. **D**. Viability of saline-treated primary murine bone-marrow neutrophils compared to neutrophils primed with GM-CSF or G-CSF for 21 hours. **E**. EC_50_ values for PIK-75 and SNS-032 as calculated from kinetic live cell imaging data of murine bone-marrow neutrophils shown in **F**., concentrations as marked. For all above, data represent mean ± SEM from three independent samples.

Using a high-throughput live cell imaging approach reported previously [15], we examined the effect of the kinase inhibitors on the viability of bone marrow neutrophils to determine their potential to cause neutropenia (Figure 2). Because G-CSF and GM-CSF are dominant regulators of granulopoiesis and neutrophil survival, and are commonly used in a clinical setting to mobilize hematopoietic stem cells into the blood and increase neutrophil production [27–31], we treated cells with these cytokines immediately prior to drug exposure. The addition of either cytokine prevents constitutive neutrophil apoptosis thereby increasing the window for detection of changes in viability induced by drug treatments.

In G-CSF-primed neutrophils, nine kinase inhibitors reduced the viability of neutrophils (Figure 2A) whereas in GM-CSF-primed neutrophils, 25 kinase inhibitors significantly reduced neutrophil viability (Figure 2B). Of the kinase inhibitors active in G-CSF-primed neutrophils, all but one was also active in the GM-CSF treated group (Figure 2C). Given that both G-CSF and GM-CSF signal through the Janus kinases (JAKs), it is intriguing that only some JAK inhibitors included in the library significantly affected neutrophil viability (AZ960, AZD1480 and AT9283), whereas others did not (CP690550, CYT387 and Merck 5). The most active compounds in this screen are the cyclin-dependent kinase (CDK) inhibitors (SNS-032 and Flavopiridol) and the PI3K p110α inhibitor PIK-75, which all induced rapid cell death in our assay system. Moreover, PIK-75 and SNS-032 induced cell death with similar potencies (Figure 2E) and kinetic profiles (Figure 2F). In contrast to other PI3 kinase inhibitors examined in this study (including PI-93, PI-103, PIK-90 and PIK-294), PIK-75 was unique in its ability to induce rapid neutrophil cell death, suggesting that off-target effects of PIK-75 are responsible for the observed toxicity (*vide infra*).

**Figure 3:**
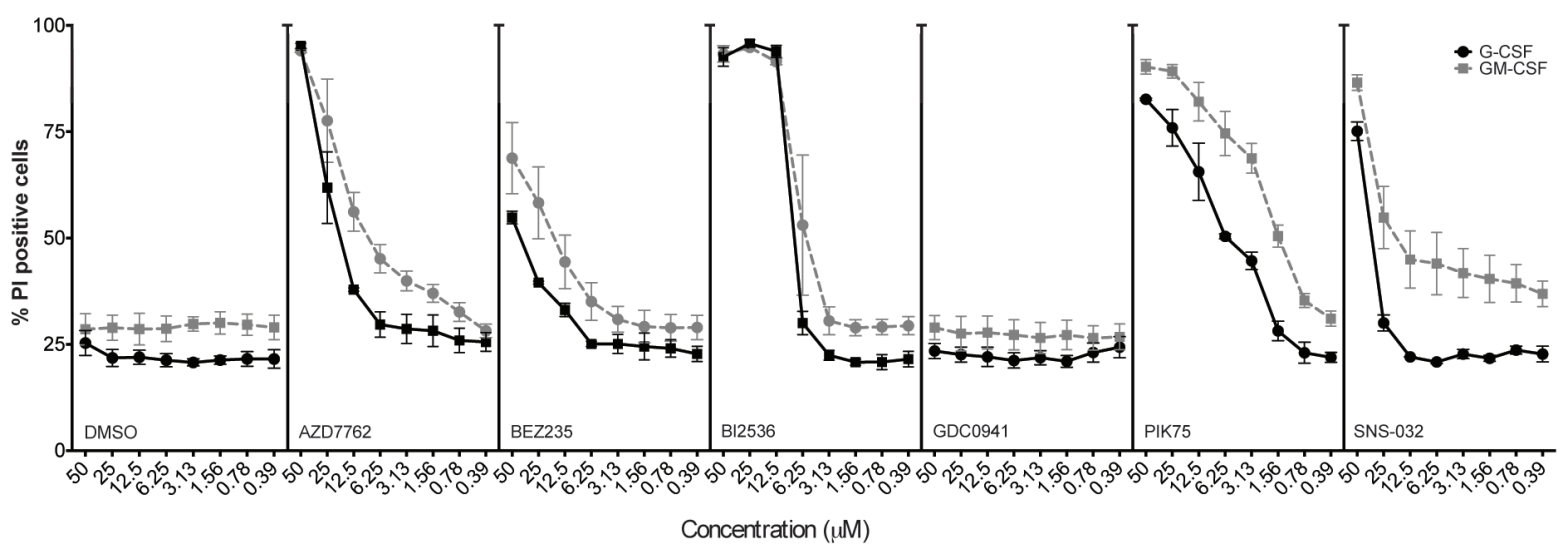
Assessment of human neutrophil viability in response to kinase inhibitor treatment Human peripheral blood neutrophils from three healthy donors were primed with G-CSF or GM-CSF for 1 hour before addition of selected kinase inhibitors (as shown) or DMSO (control) at concentrations as indicated. Neutrophil viability was assessed after 18 hours of treatment, by propidium iodide exclusion measured by flow cytometry. Mean ± SEM shown.

Human peripheral blood neutrophils from healthy donors were also studied to determine if kinase inhibitors elicit similar changes in lifespan to those of mouse neutrophils. Purified human neutrophils were primed for 1 h with G-CSF or GM-CSF, and then treated for 18 h with SNS-032, PIK-75, GDC-0941, BI2536, BEZ235, AZD7762, SB202 or SB203. In the absence of kinase inhibitors, neutrophils primed with G-CSF or GM-CSF maintained a viability of approximately 80%. Consistent with the data obtained from mouse bone marrow neutrophils, human peripheral blood neutrophils were sensitive to PIK-75 and SNS-032 but not the p38 inhibitors SB202190 and SB203580 (Figure 3). Human neutrophils were sensitive to BI2536, BEZ235 and AZD7762 however the IC50 for these inhibitors were at least 10-fold higher than for the cancer cell lines tested. Notably, human neutrophils were not sensitive to BI2536, BEZ235 and AZD7762 at the 1μM concentration used to screen mouse bone marrow neutrophils. These data suggest that similar regulatory pathways control human neutrophil lifespan and point to kinase inhibitors that offer a therapeutic window to target cancer cells while sparing bone marrow and peripheral blood neutrophils.

**Figure 4:**
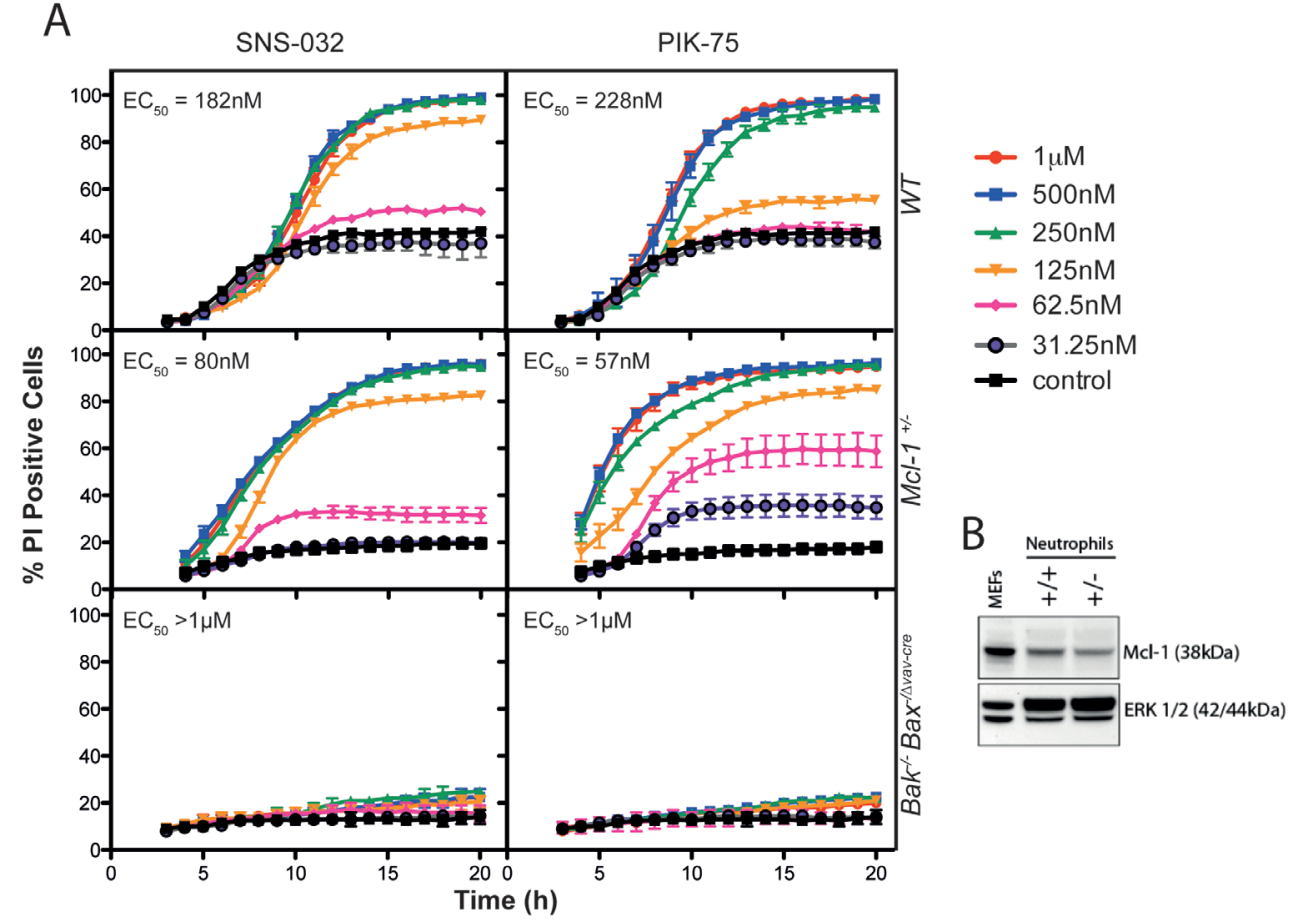
Neutrophils heterozygous for the Mcl-1 allele are more sensitive to CDK inhibition **A**. SNS-032 and PIK75 induce Bak/Bax-dependent apoptosis in primary murine bone marrow neutrophils that is sensitized by haplo-insufficiency of *Mcl-1*. Dose-dependent changes to neutrophil viability induced by PIK-75 or SNS-032 were assessed by live cell imaging over 20 hours, with genotypes and concentrations as marked. EC_50_ values were calculated from the 12 hour time-point, data represent mean ± SEM with *n* = 3 per genotype. **B**. Immunoblot of Mcl-1 protein in bone marrow neutrophils from *Mcl-1*+/- mice, and from mouse embryonic fibroblasts as a control.

To further explore the mechanisms underpinning the rapid neutrophil cell death induced by PIK-75 and SNS-032, we exposed neutrophils isolated from mice heterozygous for the *Mcl-1* allele or deficient in the dual apoptosis regulators *Bax* and *Bak*. The cytotoxicity was accelerated by the loss of a single allele of *Mcl-1* and completely abrogated by the loss of *Bak* and *Bax* (Figure 4). The loss of a single allele of *Mcl-1* reduced the EC_50_ for both SNS-032 and PIK-75 from 182 nM and 228 nM in wild-type cells to 80 nM and 57 nM in *Mcl-1*+/- cells, respectively. These data clearly indicate that neutrophil cell death induced by these compounds is mediated through the apoptotic pathway.

### Effect of compounds on hematopoietic myeloid progenitors

**Figure 5:**
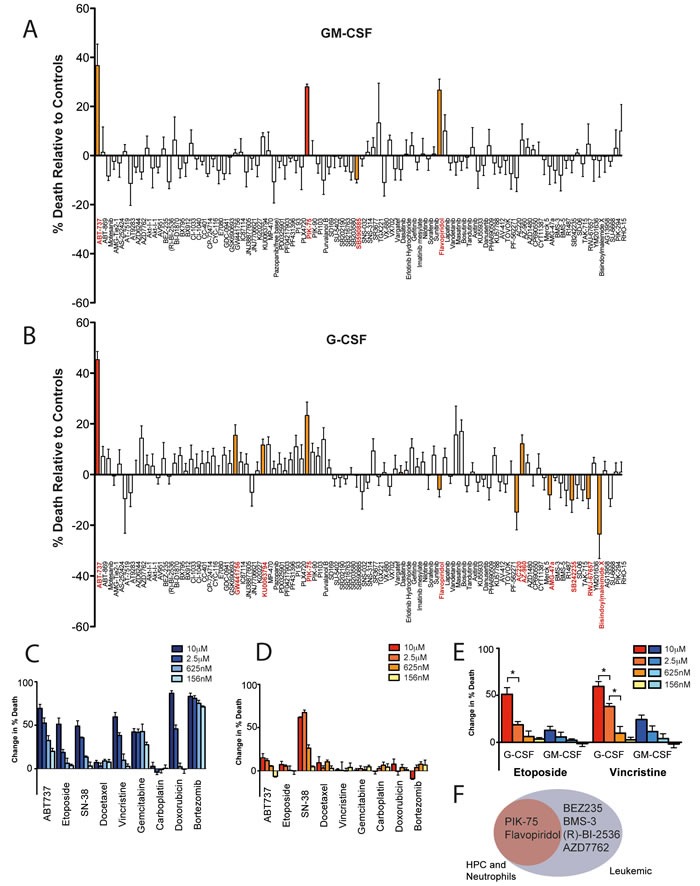
Viability of hematopoietic myeloid progenitor cells in response to kinase inhibitors or chemotherapeutics **A**. & **B**. Viability analysis from live cell imaging of primary murine myeloid HPCs primed with either GM-CSF (A) or G-CSF (B) in response to a panel of kinase inhibitors [μM] over 24 hours. Significant results (*p* < 0.05) denoted in red (when adjusted for multiple comparison testing) or orange (not adjusted for multiple comparison testing). (**C** & **D**) Dose-dependent effects of common chemotherapeutics on GM-CSF-primed primary murine myeloid HPCs **C**. or GM-CSF-primed primary murine bone-marrow neutrophils **D**. as assessed by live cell imaging analysis (concentrations as marked). **E**. Comparison of G-CSF (red/orange) or GM-CSF (blue) priming of myeloid HPCs before treatment with chemotherapeutics as assessed by live cell imaging (concentrations as marked). Bar graphs display percentage viability relative to G-CSF- or GM-CSF-only primed controls at 12 hours. Mean ± SEM with *n* = 3 independent samples. * *p* < 0.05. **F**. Venn diagram representing compounds that induce apoptosis of all leukemia cell lines tested (EC50 < 1 μM), and their apoptosis-inducing potential on hematopoietic progenitor cells and neutrophils.

The kinase inhibitor library was also profiled against hematopoietic myeloid cells (defined as lin^-^c-Kit^+^Sca-1^-^ cells) to determine the effects of these agents on cells capable of generating neutrophils. In contrast to the sensitivity of neutrophils to a broad range of kinase inhibitors, hematopoietic myeloid progenitor cells (HPC) were largely insensitive to the kinase inhibitor panel, with the exception of PIK-75, which was active against both G-CSF- and GM-CSF-treated HPC (Figure 5A and 5B). ABT-737, the small molecule antagonist of Bcl-2, Bcl-x_L_ and Bcl-w, increased the death of hematopoietic progenitor cells (Figure 5C). In contrast, ABT-737 had no impact on mouse neutrophil viability (Figure 2). Similar results were obtained for a small panel of classical chemotherapeutics. Thus, a panel of eight commonly employed cytotoxic agents in leukemia showed profound activity against hematopoietic progenitor cells (Figure 5C) whereas only SN-38, the active metabolite of irinotecan, potently decreased neutrophil viability (Figure 5D). Priming hematopoietic progenitor cells with GM-CSF, but not G-CSF, partially protected cells from the effects of vincristine and etoposide (Figure 5E).

## DISCUSSION

The discovery and development of kinase inhibitors targeted to specific kinases and kinase families has been a focus for therapeutic development for over 15 years [32, 33]. Most kinase inhibitors bind to the highly conserved ATP binding site, and the vast majority possess a spectrum of activities across the kinome [18–20, 34, 35]. Cell-based profiling of libraries of kinase inhibitors therefore offers the possibility of studying inhibition of multiple signaling networks simultaneously in an unbiased manner. The kinase inhibitory profile of active compounds from these screens, when available, can then be analysed to determine the specific kinases, or kinase combinations, driving the phenotypic response. In addition, chemoproteomic methods can be employed to identify previously unknown biological targets of active compounds [36–38]. The deconvolution of active compounds from phenotypic screens using kinase-targeted compound libraries may therefore lead to the identification of new drug targets and target combinations for specific diseases, and a number of examples of this approach have been published recently, particularly from leukemia screens [39–41]. Furthermore, this approach may also be used to identify kinases whose inhibition may lead to potential toxicity [42, 43] .

In this work we have screened a panel of kinase inhibitors against leukemia cell lines to identify compounds and kinase inhibitor classes that possess potent anti-leukemic activity. In parallel we have screened the same panel of compounds against primary mouse and human neutrophils to identify compounds and kinase inhibitory profiles that lead to neutrophil apoptosis, as a surrogate for predicting neutropenic potential. Screening the panel of kinase inhibitors against 12 leukemia cell lines identified four compounds (PIK-75, BI2536, BEZ235, AZD7762) that were potently cytotoxic to all lines examined. Thus, BI-2536, an inhibitor of polo-like kinases 1, 2 and 3 [24], and recently identified to potently inhibit the BET bromodomain BRD4 [44, 45], possessed an IC_50_ <300 nM across all cell lines examined. Given the polo-like kinases are intimately involved in cell division through association with tubulin [46], and that BRD4 inhibition can affect both cell cycle progression [47], recruitment of oncogenic transcription factors and expression of key survival proteins [48], this potent activity is unsurprising. Indeed, PLK1 is overexpressed in AML, and PLK inhibitors have been shown to possess activity in AML [49–51] and CML [52].

The potent activity of the pan PI3K/mTOR inhibitor BEZ-235 across the panel is also consistent with published data, and further supported by the activity of the recently described PI3K/mTOR inhibitors, PF-04691502 [53] and PKI-587 [54] against HL-60 cells (Figure 1C). BEZ-235 has shown activity in preclinical studies in AML [55] and has been shown to cause a decrease in Mcl-1 levels [56], a critical survival factor for AML and for neutrophils [57, 58]. Similarly, the Chk1 inhibitor AZD-7762 displayed high potency in cell lines, and selective Chk1 inhibitors have been shown to be cytotoxic to leukemia cells alone [59], or in combination with other drugs [60, 61]. The significant number of active agents against the FLT3-ITD positive AML line MV4;11, reflects the number of kinase inhibitors that possess FLT3 inhibitory activity either by design (e.g. AC220, tandutinib) or the known propensity of many kinase inhibitors to bind to FLT3 with high affinity [18]. The selective activity of MEK inhibitors GSK-1120212, PD-0325901 and CI-1040 against HL60 cells is also consistent with reported activity of MEK inhibitors in preclinical AML studies [62, 63], and indeed MEK inhibitors have been studied clinically in AML [64]. Taken together, these data clearly indicate our parallel screening approach can identify molecular targets, and indeed specific kinase inhibitors, with relevance to dose-limiting toxicities relevant to leukemia treatment.

Our parallel study exploring the effects of the kinase inhibitor library on neutrophil viability using real-time image analysis identified some surprising results. Our data clearly show that the potent CDK inhibitors flavopiridol and SNS-032 have a significant impact on neutrophil viability. That PIK-75, a nominally selective inhibitor of PI3K p110α [23], induces neutrophil death with similar kinetics to SNS-032 is consistent with recent reports showing that this compound potently inhibits CDK7 and CDK9 [65, 66]. The cell death induced by SNS-032 and PIK-75 was accelerated by the loss of a single allele of Mcl-1 and completely abrogated by the loss of Bak and Bax indicating cell death proceeds via the intrinsic apoptotic pathway. Human neutrophils express CDK7 and CDK9, which influence transcription via the phosphorylation of RNA polymerase II [67]. The abrogation of protein synthesis by CDK9 inhibitors reduces intracellular levels of short-lived proteins such as Mcl-1, a critical pro-survival protein in neutrophils whose expression level is intimately linked with neutrophil survival [68–70]. Indeed, clinical experience with SNS-032 in patients with chronic lymphocytic leukemia and multiple myeloma identified neutropenia as the dose-limiting side-effect, thought to be a consequence of decreased expression of Mcl-1 and XIAP [71]. The effect of CDK9 inhibition on neutrophils has been reported previously [67], and CDK inhibitors have been proposed as potential anti-inflammatory agents via actions on Mcl-1 [72–74]. However, the mechanism of action of CDK inhibition has been only previously inferred from analysis of Mcl-1 protein levels [67, 70, 72, 75, 76]. Using a genetic approach, we now show that the loss of Mcl-1 protein increases the sensitivity of primary bone marrow neutrophils to SNS-032 and the inhibitor PIK-75. We cannot, however, exclude a role for PI3 kinase signaling in the death-inducing effects of PIK-75 because Mcl-1 expression is also regulated in part by PI3 kinase [77], though notably other PI3K inhibitors in the kinase panel did not induce neutrophil death with the same kinetics. The effect of inhibitors of the PI3K pathway on neutrophil viability was variable. Whilst the PI3K/mTOR inhibitor BEZ-235 had minimal impact, some neutrophil toxicity was observed for the inhibitors (PI-93, PI-103, PIK-294) over the timeframe of the experiment. Other investigators have noted important roles for PI3 kinase pathways in the viability of GM-CSF-stimulated neutrophils [78] which is consistent with our data (Figure 2B). The limited effect of Jak inhibitors on neutrophil viability is also consistent with previous studies using mice with a conditional deletion of STAT3 [79, 80]. However, the cytotoxicity of the JAK inhibitors TG-101348, AT-9283 and AZD-1480 may reflect undesired off-target activity of these compounds (e.g. aurora kinase activity for TG-101348 and AT-9283; multiple targets for TG-101348 [44, 81]), and notably clinical trials of all three compounds have been discontinued.

In contrast to neutrophils, hematopoietic myeloid progenitor cells were less sensitive to the kinase inhibitor library, with the exception of PIK-75 (Figure 5), though this likely reflects the multikinase activity of this compound [66]. The small molecule antagonist of Bcl-2, Bcl-xL and Bcl-w, ABT-737, which showed minimal effects on neutrophils in our assay system, increased the death of hematopoietic myeloid progenitor cells. This is consistent with the reported role of Bcl-2 in the viability of hematopoietic progenitor cells [82]. By contrast, mature post-mitotic neutrophils were not sensitive to the panel of chemotherapeutics, with the exception of SN-38, whereas hematopoietic myeloid progenitor cells were exquisitely sensitive to these chemotherapeutics. Interestingly, although carboplatin and docetaxel induce neutropenia in the clinic [83, 84], no effects on hematopoietic myeloid progenitor cells or neutrophils were observed in these assays, suggesting that these cytotoxic drugs may induce the death of non-hematopoietic cells in the bone marrow niche or exert cytostatic effects on hematopoietic cells that impair neutrophil production.

In summary, our combined screening approach allows for the identification of specific compounds and kinase target combinations with utility in treating leukemia and lymphomas, whilst at the same time identifying whether these compounds have the potential to cause neutropenia through toxicity to neutrophils and their progenitors. Further, the kinase inhibitory properties of the compounds employed provides an opportunity to identify the dominant pathways controlling neutrophil viability. Our data demonstrates that compounds with significant CDK9 (and CDK7) activity are likely to be toxic to neutrophils. Conversely, PLK inhibitors and those targeting PIK3/mTOR and separately MEK and Chk1, are likely to have minimal neutropenic potential whilst having considerable activity against leukemias.

## MATERIALS AND METHODS

### Cell lines and culture

Leukemia and lymphoma cell lines were grown in RPMI containing 10% FCS. Cells were obtained from ATCC (HEL 92.1.7, Jurkat, MEG01, THP-1, MV4;11, KU-812, U937), the NCI (K562, HL-60, MOLT-4) or DSMZ (EOL-1, OCI-AML3) and used at low passage. Cell lines are routinely tested for mycoplasma and genotyped by short tandem repeating (STR) profiling to confirm identity.

### Cell proliferation assay

Compounds were obtained from SynMedChem and dissolved at 10 μM in DMSO. Serial 2-fold dilutions of each compound were prepared in DMSO using a liquid handling robot (Janus, Perkin Elmer). Each dilution (100nL) was transferred to duplicate tissue culture plates containing 50 μL of media containing 5 × 10^3^ cells using a 384 well pin tool. Cells were incubated for 48 h and cell viability was determined using Cell Titer Glow reagents as described by the manufacturer (Promega). Data was acquired using a luminescent plate reader (Envision, Perkin Elmer) and analysed using Activity Base (IDBS, Guilford, UK) and visualised using Spotfire (TIBCO, Palo Alto, CA) as described previously [14]. Each cell line was assayed in independent experiments on at least two occasions. The activity of PI3K/mTor and MEK inhibitors in HL-60 cells were validated using Cell Titer Glo assays in 96-well plates. Serial 2-fold dilutions of each compound were prepared in media containing 0.2% DMSO and 50 μl of each dilution was added to 10^4^ cells in 50 μl of media. Cells were incubated for 48 h and cell viability was determined using Cell Titer Glow assay as described above.

### Mice

C57BL/6J, *Mcl1*+/^-^, *Bak*-/- *Bax*-/ΔvavCre and littermate controls on a C57BL/6J background were bred at the Walter and Eliza Hall Institute and analyzed at 6-10 weeks of age after CO_2_ asphyxiation. All experiments were carried out in accordance with institutional animal ethics guidelines and approval.

### Neutrophil preparation

Mouse bone marrow neutrophils were prepared as described [15]. Neutrophils were resuspended in phenol red-free DMEM/10% FBS for imaging studies. The purity of neutrophil preparations was routinely 96% ± 4% (Mean ± SD, *n* = 50) as assessed by cytology following May-Grünwald/Giemsa staining.

### Myeloid progenitor cell preparation

For purification of myeloid progenitor cells, bone marrow cells were collected in DMEM/10% FCS. After red cell lysis, cells were stained with biotin-conjugated antibodies against Ter119, Gr-1, Mac-1, B220, CD4, CD8 and IL-7Rα and incubated with streptavidin-microbeads (Miltenyi Biotech). Lineage positive cells were depleted by magnetic separation using MACS LS columns and QuadroMACS^TM^ separator magnets (Miltenyi Biotech). Hematopoietic myeloid progenitor cells that were propidium iodide (PI) negative, lineage-negative, Sca-1 negative and c-Kit positive, were sorted and collected by flow cytometry on a FACSAria or Diva (BD Biosciences). For imaging studies, cells were resuspended in StemPro-34 SFM (Invitrogen). Cells were cultured in 10 ng/mL human G-CSF (Neupogen, Amgen), 10 ng/mL recombinant mouse GM-CSF or 10 ng/mL recombinant human GM-CSF.

### Human peripheral blood neutrophils

Peripheral blood was obtained from normal healthy donors in 2015 after obtaining informed written consent under an approved institutional review board protocol at Boston Children's Hospital. Donors were 18 years of age or older and had not taken anti-inflammatory medications during the 2 weeks before donating. Red blood cells were removed from 5 mL blood by hypotonic lysis in 45 mL 0.168 M NH_4_Cl, 11.9 mM NaHCO_3_, 10 μM EDTA, pH7.3 on ice before immediate centrifugation at 400 × g for 5 min. Human neutrophils were then isolated on a three-layer Percoll gradient as described for mouse bone marrow neutrophils as described [16]. The purity of neutrophil preparations was 93 ± 1% (mean ± SD, *n* = 3) as assessed by May-Grünwald Giemsa staining. Contaminating cells were comprised of eosinophils (7 ± 1%). Neutrophils were resuspended in DMEM/10%FBS, and primed with 10 ng/mL recombinant human G-CSF (Amgen) or 10 ng/mL recombinant human GM-CSF (Leukine, Sargramostim) for 1 h. Compounds or DMSO carrier were added for 16 h. Viability was assessed by staining neutrophils with propidium iodide and analyzing on a BD Fortessa.

### Compounds

Kinase inhibitors were purchased from SYN kinase (Melbourne, Australia) and were used at 1 μM, unless otherwise specified, with dilutions performed in assay buffer containing 0.1% DMSO. Chemotherapeutics, diluted in PBS immediately prior to treatment, were obtained from the following suppliers: ABT-737 (AbbVie); Bortezomib (Janssen); Carboplatin, Doxorubicin, Etoposide, Vincristine (DBL); Docetaxel (Aventis); Gemcitabine (Ebewe, InterPharma); and, 7-ethyl-10-hydroxycamptothecin (SN-38) (LKT Laboratories).

### Live cell imaging and quantitative analysis

Cells were labeled with 160 nM Cell Tracker Green (Invitrogen) and plated in 96- or 384-well optical bottom assay plates (Nunc, BD) at 10^5^ or 1.5 × 10^3^ cells per well, respectively. Viability was monitored using 2 μg/mL PI (Sigma). Images were captured every 30-60 min by the Zeiss Axiovert 200M Microscope at 37 °C/10% CO_2_. Quantitative analysis was performed using a custom-scripted MetaMorph (Molecular Devices) journal suite incorporating the count nuclei function, reconstruction & subtraction functions and Integrated Morphometry Analysis to select and analyze cells of interest. The MetaMorph script is available upon request.

### Statistical analysis

Unless otherwise specified, data are presented as mean ± SEM of *n* = 3. Where *n* = 6, comparisons were made using a statistical modeling package CompareGrowthCurves [17]. Comparisons were performed using a Student's t-test and adjusted by Hommel's Modification of the Bonferroni t test.
